# Disparities in Rates of Inpatient Mortality and Adverse Events: Race/Ethnicity and Language as Independent Contributors

**DOI:** 10.3390/ijerph111213017

**Published:** 2014-12-12

**Authors:** Anika L. Hines, Roxanne M. Andrews, Ernest Moy, Marguerite L. Barrett, Rosanna M. Coffey

**Affiliations:** 1 Truven Health Analytics, 7700 Old Georgetown Road Suite 650, Bethesda, MD 20814, USA; E-Mail: rosanna.coffey@truvenhealth.com; 2 Agency for Healthcare Research and Quality, 540 Gaither Road, Rockville, MD 20850, USA; E-Mails: roxanne.andrews@ahrq.hhs.gov (R.M.A.); ernest.moy@ahrq.hhs.gov (E.M.); 3 M.L. Barrett, Inc., 13943 Boquita Drive, Del Mar, CA 92014, USA; E-Mail: barrettm@earthlink.com

**Keywords:** health status disparities, language, inpatients, quality indicators, Whites, Blacks, Asians, Hispanics

## Abstract

Patients with limited English proficiency have known limitations accessing health care, but differences in hospital outcomes once access is obtained are unknown. We investigate inpatient mortality rates and obstetric trauma for self-reported speakers of English, Spanish, and languages of Asia and the Pacific Islands (API) and compare quality of care by language with patterns by race/ethnicity. Data were from the United States Agency for Healthcare Research and Quality, Healthcare Cost and Utilization Project, 2009 State Inpatient Databases for California. There were 3,757,218 records. Speaking a non-English principal language and having a non-White race/ethnicity did not place patients at higher risk for inpatient mortality; the exception was significantly higher stroke mortality for Japanese-speaking patients. Patients who spoke API languages or had API race/ethnicity had higher risk for obstetric trauma than English-speaking White patients. Spanish-speaking Hispanic patients had more obstetric trauma than English-speaking Hispanic patients. The influence of language on obstetric trauma and the potential effects of interpretation services on inpatient care are discussed. The broader context of policy implications for collection and reporting of language data is also presented. Results from other countries with and without English as a primary language are needed for the broadest interpretation and generalization of outcomes.

## 1. Introduction

The role of a patient’s principal spoken language in access to and receipt of primary and preventive health care has been well documented. Patients with limited English proficiency tend to have lower rates of health insurance coverage and typically lack a usual source of care [1]. In addition, patients with English as a second language may be managed differently by health care professionals and receive fewer recommended health care services than native English speakers, regardless of their level of fluency [2,3,4]. Studies also indicate that speakers of English as a second language report lower levels of satisfaction with emergency care [5] and with timeliness, provider communication, and staff helpfulness in other health care settings [6]. 

Fewer studies have examined the effect of language barriers on quality of care and related outcomes—including adverse medical events and inpatient mortality—once physical access to health care is achieved. Language barriers have been identified as key contributors to adverse medical events [7]. Although language does not appear to play a role in inpatient mortality [8,9,10], language barriers may [8,11] or may not [10] prolong the length of hospital stay or increase readmissions [10], depending on the condition. One study acknowledged that increased length of stay may be attributed to other hospital-level factors rather than language barriers, but the evidence is uncertain [9]. 

The studies to date have been limited to a few hospitals within a system, have focused on a single condition, or have broadly characterized the patient’s language as “English *vs.* non-English” without accounting for the specific non-English language spoken. The constraints of these studies and their variable results reflect the paucity of viable language data currently being collected and reported, and they prohibit the ability to draw conclusions about what may be a significant barrier to the provision of health care. 

The effects of a patient’s language and race/ethnicity on health care have gained increasing attention among health policy audiences in the United States, particularly in light of emerging federal regulations related to the Affordable Care Act and other government policies. The need for additional research is critical and timely. Health care leaders in other countries can benefit from the extensive amount of data available to inform this project. 

The goals of the present study are to examine the rates of inpatient mortality and adverse events across language groups—English, Spanish, and languages of Asia and the Pacific Islands (API)—and to compare patterns of variation in quality of care by language with patterns by race and ethnicity. 

## 2. Methods

### 2.1. Data Source 

We used data from the United States Agency for Healthcare Research and Quality (AHRQ), Healthcare Cost and Utilization Project, 2009 State Inpatient Databases for the State of California. The databases include discharge abstracts that contain demographic and clinical information for all inpatient stays in all hospitals in the state [12]. California represents a large population, and it is one of the few states that collects and reports data on the patient’s principal language in addition to race and ethnicity. Beginning in 2009, hospitals in California reported the principal language the patient uses in communicating with those in the health care community [13]. Data submitted by facilities are standardized into language categories. Less than 3 percent of California records were coded as having an “unknown” principal language in 2009.

### 2.2. Study Population 

The study population included all adult inpatients admitted to community, non-rehabilitative hospitals in California for selected discharges. Within this group, we chose adult discharge data based on the population criteria for specific AHRQ Quality Indicators (QIs) examined [14]. AHRQ Inpatient Quality Indicators (IQIs) reflect quality of care for adults inside hospitals, including inpatient mortality for medical conditions and surgical procedures [15]. AHRQ Patient Safety Indicators (PSIs) reflect quality of care for adults inside hospitals [16]. They focus on potentially avoidable complications and iatrogenic events. Many of the QIs have been endorsed by the National Quality Forum for public reporting on hospital performance. To assure sufficient statistical power and reliability, we focused on IQIs and PSIs with the largest sample size: adult inpatient mortality for five medical conditions—acute myocardial infarction (AMI), congestive heart failure (CHF), stroke, gastrointestinal (GI) hemorrhage, and pneumonia—and two obstetric trauma measures—vaginal delivery *with* and *without* instrument assistance. 

We then described inpatient discharges by the patient’s self-reported principal language and self-reported race/ethnicity. We reported the patient’s language as presented in the California data for English and Spanish and combined the languages spoken by various API subpopulations into a category “API languages” to increase the sample size and statistical power for this group. API languages included Chinese, Japanese, Vietnamese, Tagalog, Korean, Thai, Lao, Mandarin, Cantonese, Hmong, Ilocano, Iu Mien, Indonesian, Mon-Khmer, Tonga, Hindi, Urdu, Burmese, Telugu, Bengali, Tamil, Gujarati, Panjabi, Malayalam, Marathi, and Kannada. We excluded individuals whose principal language was not among those listed because of insufficient numbers for analysis.

The selection process resulted in 3,757,218 inpatient records in the following principal language categories: English speakers (n = 3,211,939; 85.5%); Spanish speakers (n = 474,267; 12.6%); and API language speakers (n = 71,495; 1.9%). 

### 2.3. Demographic Characteristics 

We used patient demographic data elements from the State Inpatient Databases to describe the study population: age, gender, race/ethnicity (Non-Hispanic White, non-Hispanic Black, Hispanic, non-Hispanic API, or Other), expected primary payer (Medicare, Medicaid, private insurance, self-pay/no charge), median household income of the patient’s ZIP Code of residence (in quartiles), and urban-rural location of the patient’s residence. The urban-rural measure uses the National Center for Health Statistics categorizations: large central metropolitan (central counties with metropolitan service areas of 1 million or more population); large fringe metropolitan (fringe counties with metropolitan service areas of 1 million or more population); medium metropolitan (counties with metropolitan service areas of 250,000–999,999 population); small metropolitan (counties with metropolitan service areas of 50,000–249,999 population); micropolitan (counties with a city of 10,000 or more population); and not metropolitan or micropolitan (counties without a city of 10,000 or more population) [17].

### 2.4. Data Analyses 

We conducted a descriptive analysis of IQI and PSI rates per 1000 admissions and per 1000 vaginal deliveries, respectively, across race/ethnicity, principal language, and race/ethnicity–language groups using risk adjustments provided in the AHRQ QI Software Version 4.1. The mortality IQIs were adjusted for age, gender, age-gender interactions, and patient’s clinical condition using major diagnostic categories and the risk of mortality score from the All Patient Refined Diagnosis Related Groups (APR-DRGs). In determining the risk of mortality subclass (minor, moderate, major, and extreme) the APR-DRG software examines the risk of mortality level for each secondary diagnosis that is unrelated to the principal diagnosis and factors in age, APR-DRG, operating room and non-operating-room procedures, and interactions of multiple secondary diagnoses. We did not use the present-on-admission data element. The rates of obstetric traumas during vaginal deliveries with and without instrument assistance were adjusted by age. 

The analyses compared the selected IQI and PSI risk-adjusted rates across languages and across languages within racial/ethnic groups. Where adequate sample sizes were available, we also reported estimates for API languages separately. We used the risk-adjusted rates and corresponding standard errors to calculate the *t*-test statistic (two-sided) and *p*-value. 

## 3. Results

### 3.1. Demographic Characteristics 

The demographic characteristics of inpatient discharges (children and adults) by principal language spoken are displayed as point estimates in Table 1. Patients speaking API languages had the highest average age (58 years) compared with patients speaking Spanish (35 years) and English (46 years). The most common type of insurance coverage varied with the patient’s principal language: Medicare (46.1%) for patients speaking API languages; Medicaid (57.8%) for patients speaking Spanish; and private insurance (37.3%) or Medicare (33.4%) for patients speaking English. Patients speaking API languages and English most frequently lived in the highest income communities (40.3% and 36.0%, respectively); patients speaking Spanish most frequently lived in the lowest income communities (31.6%). 

**Table 1:** Characteristics of inpatient discharges by principal language spoken, 2009.

Characteristic	Principal Language Spoken										
	English	Spanish	Languages of API								
			Total	Chinese	Hindi	Japanese	Korean	Other	Tagalog	Thai	Vietnamese
Total discharges (N = 3,757,218)	3,211,457	474,267	71,495	24,643	2592	2608	11,980	773	12,134	674	16,089
Average age (years)	46.0	35.4	58.4	59.0	48.5	57.6	57.9	44.8	68.0	53.9	53.1
**Gender (%)**											
Female	58.0	63.9	61.6	60.2	64.2	70.3	64.2	67.4	61.7	68.4	59.4
Male	42.0	36.1	38.4	39.8	35.8	29.7	35.8	32.6	38.3	31.6	40.6
**Expected primary payment source (%)**											
Private insurance	37.3	14.2	24.0	23.1	43.4	43.2	22.6	39.0	20.3	30.5	22.0
Medicare	33.4	18.0	46.1	49.0	25.8	47.1	47.1	21.2	53.3	27.9	40.5
Medicaid	21.3	57.8	21.6	17.7	24.1	5.3	19.0	32.3	21.2	29.4	31.1
Self-pay or no charge	3.3	4.7	5.0	8.1	3.5	3.2	7.8	3.5	1.8	5.6	1.3
**Median household income of patient’s ZIP Code (%)**											
First quartile (lowest income)	16.0	31.6	15.3	11.0	6.9	11.5	43.0	9.4	12.4	23.7	5.4
Second quartile	18.9	24.7	14.6	20.5	14.6	12.1	8.0	14.6	9.3	16.3	14.7
Third quartile	29.1	29.1	29.9	28.5	17.5	28.0	21.9	24.5	27.1	27.8	42.6
Fourth quartile (highest income)	36.0	14.6	40.3	40.0	60.9	48.4	27.0	51.5	51.3	32.3	37.3
**Location of patient’s residence (%)**											
Large central metropolitan	62.1	69.8	89.8	93.5	74.3	86.4	95.4	73.1	75.2	85.4	95.1
Large fringe metropolitan	13.3	8.3	6.6	4.9	8.9	8.0	3.0	11.3	17.8	8.7	2.8
Medium metropolitan	17.7	17.2	3.0	1.3	11.0	4.1	1.4	15.1	6.3	4.8	2.0
Small metropolitan	4.1	4.0	0.5	0.2	5.5	1.2	0.2	DSU	0.5	DSU	0.1
Micropolitan	2.0	0.5	0.1	DSU	DSU	DSU	DSU	DSU	0.1	DSU	DSU
Not metropolitan or micropolitan	0.8	0.3	0.0	0.06	DSU	DSU	DSU	DSU	DSU	DSU	DSU
**Race/ethnicity (%)**											
White	57.0	2.7	2.0								
Hispanic	22.2	96.1	1.8								
API	7.3	0.1	93.2								
Black	10.2	0.1	0.2								
Other	3.2	1.0	2.9								

Notes: Individuals represented in the White, API, and Black racial/ethnic categories are non-Hispanic. Cells with a frequency of records <10 are not displayed. API = Asia and the Pacific Islands; DSU = data statistically unreliable (data do not meet the criteria for statistical reliability, data quality, or confidentiality). **Source:** Healthcare Cost and Utilization Project, State Inpatient Databases, California community, non-rehabilitation hospitals, 2009 [12,13].

**Table 2:** Characteristics of Hispanic and Asian/Pacific Islander inpatient discharges by patient race/ethnicity and principal language, 2009.

Characteristic	Hispanic Patient, English Language	Hispanic Patient, Spanish Language	API Patient, English Language	API Patient, API Language
Total discharges (n)	714,365	455,625	234,642	66,609
Average age (years)	30.8	35.5	39.2	58.5
**Gender (%)**				
Female	60.4	64.0	61.1	61.5
Male	39.6	36.0	38.9	38.5
**Expected primary payment source (%)**				
Private insurance	33.9	14.0	55.5	23.9
Medicare	16.0	18.0	22.7	46.6
Medicaid	40.6	57.9	16.2	21.3
Self-pay/No charge	4.1	4.8	2.5	5.0
**Median income of patient’s ZIP Code (%)**				
First quartile (lowest income)	22.7	31.7	10.0	15.5
Second quartile	24.2	24.8	12.2	14.5
Third quartile	31.8	29.1	26.8	30.0
Fourth quartile (highest income)	21.4	14.4	51.0	40.0
**Location of patient’s residence (%) **				
Large central metropolitan	64.2	70.2	76.5	90.4
Large fringe metropolitan	12.4	8.3	11.4	6.5
Medium metropolitan	18.9	16.8	10.5	2.6
Small metropolitan	3.8	4.0	1.2	0.4
Micropolitan	0.4	0.4	0.3	0.0
Not metropolitan or micropolitan	0.2	0.3	0.1	0.0

Notes: Individuals represented in the API racial/ethnic category are non-Hispanic. API = Asia and the Pacific Islands. **Source**: Healthcare Cost and Utilization Project, State Inpatient Databases, California community, non-rehabilitation hospitals, 2009 [12,13].

**Table 3:** Risk-adjusted mortality and obstetric trauma rates for Hispanic and Asian/Pacific Islander speakers by language compared with White speakers of English, 2009.

Characteristic	Acute Myocardial Infarction, Inpatient Mortality, Mean (SD)	Congestive Heart Failure, Inpatient Mortality, Mean (SD)	Stroke, Inpatient Mortality, Mean (SD)	Gastro-Intestinal Hemorrhage, Inpatient Mortality, Mean (SD)	Pneumonia, Inpatient Mortality, Mean (SD)	Obstetric Trauma, Instrument-Assisted Deliveries, Mean (SD)	Obstetric Trauma, Unassisted Deliveries, Mean (SD)
**White**							
English speakers (ref ^a^)	59.07 (1.26)	28.03 (0.66)	86.26 (1.33)	21.58 (0.81)	34.57 (0.68)	119.62 (3.55)	22.40 (0.51)
**Hispanic**							
English speakers (ref ^b^)	56.91 (3.19)	**22.55 (1.69)** **^c^**	80.81 (2.84)	18.09 (1.81)	34.37 (1.72)	**92.65 (3.91)** **^c^**	**14.98 (0.42)** **^c^**
Spanish speakers	60.42 (3.66)	**18.55 (1.70)** **^c^**	**74.69 (3.20)** **^c^**	17.42 (2.13)	**28.19 (1.80)** **^b,c^**	**98.66 (4.27)** **^c^**	**18.59 (0.55)** **^c,d^**
**Asian and Pacific Islander**							
English speakers (ref ^b^)	62.38 (3.87)	**21.98 (2.23)** **^c^**	**76.85 (3.45)** **^a^**	19.63 (2.58)	30.82 (2.21)	**167.72 (7.49)** **^c^**	**34.44 (1.29)** **^c^**
API speakers	56.61 (5.60)	**19.06 (2.78)** **^c^**	77.47 (4.74)	23.04 (3.36)	33.36 (2.59)	**165.02 (14.99)** **^c^**	**50.01 (8.67)** **^c^**

Notes: ^a^ *p* < 0.05 compared with White, English speakers; ^b^ *p* < 0.05 compared with English speakers of same ethnicity; ^c^ *p* < 0.01 compared with White, English speakers; ^d^ *p* < 0.01 compared with English speakers of same ethnicity. Values in bold represent statistically significant differences relative to indicated references. Risk-adjusted rate per 1,000 admissions or 1000 vaginal deliveries (SE). Individuals represented in the White and API racial/ethnic categories are non-Hispanic. API = Asia and the Pacific Islands; IQI = Inpatient Quality Indicator; PSI = Patient Safety Indicator; ref = reference for statistical tests across languages within race/ethnicity. Source: Healthcare Cost and Utilization Project, State Inpatient Databases, California community, non-rehabilitation hospitals, 2009, and version 4.1 of the IQI and PSI software. IQIs are adjusted by age, gender, major diagnostic category, and APR-DRG risk of mortality. PSIs are adjusted by age [12,13].

Although the majority of all patients resided in large central metropolitan areas, the distribution varied: 89.8% of patients speaking API languages, 69.8% of patients speaking Spanish, and 62.1% of patients speaking English. 

We also examined the demographic characteristics of all Hispanic and API inpatient discharges by ethnic group to determine differences between English and non-English speakers (Table 2). Spanish-speaking Hispanic patients were older, on average, (35.5 years) than English-speaking Hispanic patients (30.8 years). Spanish-speaking Hispanic patients also were more likely to be insured by Medicaid and to reside in the lowest income communities compared with English-speaking Hispanic patients. API inpatients who spoke an API language were older (58.5 years) than English-speaking API inpatients (39.2 years). API inpatients who spoke an API language were more likely to be insured by Medicare and less likely to live in the highest income communities than English-speaking API patients. 

### 3.2. Risk-Adjusted Hospital Outcomes by Language and by Race/Ethnicity 

**Figure 1:**
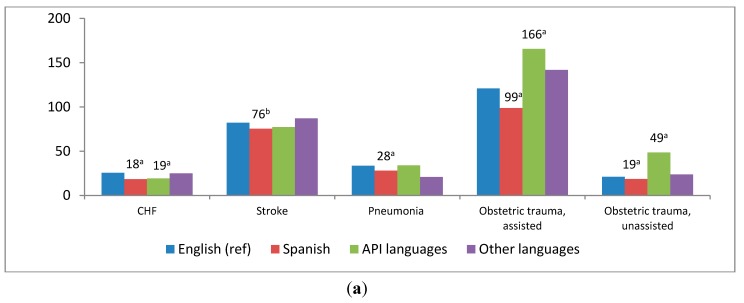

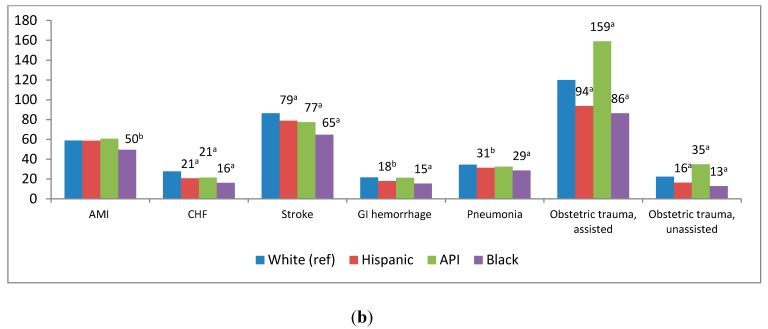
Risk-adjusted inpatient mortality for common conditions and obstetric trauma rates by language and race/ethnicity, 2009.

Notes: ^a^ *p* < 0.01; ^b^ *p* < 0.05. Labeled values on graphic represent statistically significant differences relative to indicated reference. Individuals represented in the White, API, and Black racial/ethnic categories are non-Hispanic. Risk-adjusted rate per 1000 admissions or 1000 vaginal deliveries. API = Asia and the Pacific Islands; ref = reference group for statistical tests with race/ethnicity or within language. **Source:** Healthcare Cost and Utilization Project, State Inpatient Databases, California community, non-rehabilitation hospitals, 2009, and version 4.1 of the IQI and PSI software. IQIs are adjusted by age, gender, major diagnostic category, and APR-DRG risk of mortality. PSIs are adjusted by age [15,16].

In general, risk-adjusted inpatient mortality for the selected medical conditions among speakers of Spanish and API languages was similar to (or lower than) that of English-speaking patients. Age-adjusted rates of obstetric trauma were lower among speakers of Spanish and higher among speakers of API languages relative to English speakers (Figure 1a). 

Comparisons of racial/ethnic characteristics yielded patterns similar to those observed in the language analysis (Figure 1b). In general, rates of mortality and obstetric trauma were lower for racial/ethnic minority groups than for White patients. The only exception to this pattern was higher rates of obstetric trauma in instrument-assisted (159.05 *vs.* 119.94; *p* < 0.01) and unassisted (34.76 *vs.* 22.42; *p* < 0.01) deliveries among Asians and Pacific Islanders compared with White patients. 

**Figure 2:**
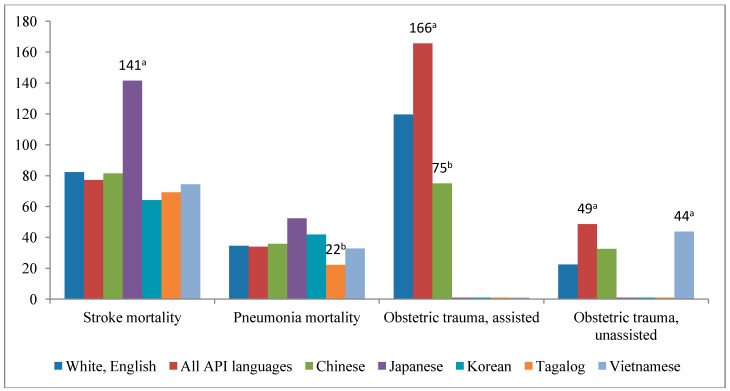
Risk-adjusted inpatient mortality and obstetric trauma rates by Asian/Pacific Island language subgroups compared with White speakers of English, 2009.

Notes: ^a^ *p* < 0.01; ^b^ *p* < 0.05; Labeled values on graphic represent statistically significant differences relative to indicated reference. Individuals represented in the White and API racial/ethnic categories are non-Hispanic. Risk-adjusted rate per 1000 admissions or 1,000 vaginal deliveries. API = Asia and the Pacific Islands; DSU = data statistically unreliable (data do not meet the criteria for statistical reliability, data quality, or confidentiality) for Japanese-, Korean-, Tagalog-, and Vietnamese speakers for the assisted trauma measure and for Japanese-, Korean-, and Tagalog speakers for the unassisted obstetric trauma measure; ref=reference group for statistical tests across languages. **Source:** Healthcare Cost and Utilization Project, State Inpatient Databases, California community, non-rehabilitation hospitals, 2009, and version 4.1 of the IQI and PSI software. IQIs are adjusted by age, gender, major diagnostic category, and APR-DRG risk of mortality. PSIs are adjusted by age [15,16]

We compared risk-adjusted mortality and obstetric trauma for English-speaking, non-Hispanic White patients with API subgroups that had a sample size larger than 600: Chinese, Hindi, Japanese, Korean, Tagalog, Thai, and Vietnamese (Figure 2). Overall, API language speakers demonstrated no difference in risk-adjusted inpatient stroke mortality than English speakers; however, Japanese speakers had a significantly higher risk-adjusted stroke mortality rate (141.46 *vs.* 86.38; *p* < 0.01) compared with English-speaking, non-Hispanic White patients. Individuals who spoke Tagalog demonstrated lower risk-adjusted rates of pneumonia inpatient mortality (22.22 *vs.* 34.46; *p* < 0.05) than English-speaking, non-Hispanic White patients. Age-adjusted obstetric trauma without instrument assistance was higher among Vietnamese mothers compared with English-speaking, non-Hispanic White mothers (43.76 *vs.* 22.40; *p* < 0.01). There were no statistically significant differences in rates across other API language subgroups.

### 3.3. Risk-Adjusted Hospital Outcomes within Race-Ethnicity Group by Language 

Table 3 contains risk-adjusted mortality and obstetric trauma rates for Hispanic and API patients by their spoken language, compared with non-Hispanic, White speakers of English. There were no statistically significant differences between Hispanic patients who spoke English and Hispanic patients who spoke Spanish or between API patients who spoke English and API patients who spoke an API language in inpatient risk-adjusted mortality for four of the medical conditions (AMI, CHF, stroke, and GI hemorrhage) or for obstetric trauma among vaginal deliveries with instrument assistance. However, there were differences in pneumonia mortality and obstetric trauma among instrument-assisted deliveries. Spanish-speaking Hispanic patients had lower rates of pneumonia inpatient mortality than their English-speaking counterparts (28.19 *vs.* 34.37; *p* < 0.05) and higher rates of obstetric trauma among vaginal deliveries without instrument assistance (18.59 *vs.* 14.98; *p* < 0.01). 

## 4. Discussion

Unlike the clear influence of principal language on access to care, language appears to play a varying role in hospital outcomes after access is achieved. The findings of this study agree with previous literature using smaller samples [8,9,10] in showing that individuals who speak a principal language other than English do not have higher rates of inpatient mortality than their English-speaking counterparts. In general, non-English speakers demonstrated similar or lower rates of inpatient mortality for AMI, CHF, stroke, pneumonia, and GI hemorrhage. The primary negative influence of language was a higher rate of obstetric trauma for patients speaking API languages. 

The patterns of significant differences in outcomes across race/ethnicity nearly mirrored those for language. Patients from minority groups consistently had similar or, more commonly, lower rates of inpatient mortality compared with White patients. Japanese patients had the only significantly higher stroke mortality rate. Lower rates of inpatient mortality for racial/ethnic minorities are consistent with previous studies of mortality at the Veterans Affairs (VA) hospitals [18,19,20] and non-VA hospitals in California and Pennsylvania [20]. In the present study, obstetric trauma was the only other negative outcome related to the patient’s race/ethnicity; API patients and Spanish-speaking Hispanic patients had significantly higher rates of obstetric trauma compared with White patients. 

It is tempting to conclude that neither principal language nor race/ethnicity has a deleterious effect on hospital outcomes, with a few exceptions (most notably for obstetric trauma). However, some trends observed in the outcomes suggest that race/ethnicity may be a stronger influence than language for these outcomes. As demonstrated in Figure 1a,b, eight out of 21 comparisons between speakers of English and speakers of other languages reached statistical significance, whereas 17 out of 21 comparisons between White patients and Black, Hispanic, or API patients reached statistical significance. At the very least, these differences indicate the importance of continuing to examine both language and racial/ethnic influences as data are expanded.

Lower mortality rates for non-English speakers and racial/ethnic minorities in this study could indicate that patients are not being stigmatized and are receiving the services they need once they are in the hospital environment. However, other factors not measured in this study also could contribute to these findings, such as the seemingly protective health effects experienced by recent immigrants [21]. Although Hispanic English speakers demonstrated rates of pneumonia mortality that were similar to White English speakers, Spanish-speaking Hispanic patients demonstrated significantly lower rates than White English speakers and their Hispanic English-speaking counterparts, implying possible cultural differences. Spanish-speaking Hispanic patients also had more Medicaid coverage and came from communities with the lowest median income. Race and ethnicity represent a complex interaction of genetic and cultural backgrounds [4,22]. Culturally driven differences such as the propensity to use hospitals in serious medical circumstances *vs.* dying at home, support from English-speaking family members, or other dietary, religious, or lifestyle variations cannot be discounted as factors that may influence any study involving categorization of race and ethnicity. 

### 4.1. Obstetric Trauma 

API patients had higher rates of obstetric trauma than White patients in the language and racial/ethnic analyses. Previous studies have identified Asian ethnicity as an independent risk factor for obstetric trauma, particularly severe perineal trauma. Wheeler and colleagues [23] conducted a comprehensive review of the 2000–2010 literature on this topic. The authors found that anatomical variations such as a shorter perineum do not appear to be a primary contributor. Additionally, they found that outcomes for Asian women are only poorer when they give birth in non-Asian countries (primarily Australia, the United Kingdom, and the United States). They summarize possible contributing factors mentioned by researchers as labor and birthing management techniques, communication, cultural differences, acculturation, fear, or influence of the birth attendant (particularly the attendant’s experience level). Sentell and colleagues [24] examined 5 years of statewide API data in Hawaii. Compared with White patients, Japanese, Filipino, and other Pacific Islanders had significantly higher overall delivery complication rates, whereas Native Hawaiians had significantly lower rates. Subgroup differences were also observed for obstetric trauma with and without instruments and Cesarean deliveries. 

The influence of the patient’s principal language on obstetric trauma was mentioned in early studies [25] but largely ignored in later years. Mother-practitioner communication during the birthing process is a determinant of serious injury *vs.* safe delivery. Dahlen and colleagues [26] found that severe perineal trauma occurred more often when language interpreters were needed. Our within racial/ethnic group results show higher obstetric trauma rates among Spanish-speaking Hispanic patients and suggest that the influence of language should be investigated further. Comparing API and Spanish speakers with their English-speaking ethnic counterparts reduces the likelihood that observed differences are attributable to biological or physiological features associated with race/ethnicity and highlights linguistic, cultural, procedural, or social factors related to the health care encounter as more likely contributors. The Partnership for Patients encourages communication with patients to reduce adverse events such as obstetric trauma [27]. 

### 4.2. Collection and Reporting of Language Data 

The large volume of records in this study provides more robust estimates than previous studies and leads us to consider additional data issues in studies of principal language. The aggregation of individuals of varying ranges of proficiency and from multiple cultural or ethnic groups into a single “English” category is one challenge. Although nearly 43 percent of Californians report “speaking a language other than English” [28], only about 17 percent reported a non-English language as “the principal language the patient primarily uses in communicating with those in the health care community”—the definition used in this study. This 26-point difference in proportions may be an artifact rooted in the characteristics of those who are admitted to the hospital relative to the general California population; however, it could demonstrate a true difference in how individuals report their language preferences at home *vs.* in the health care setting. Moreover, motivations behind a potential difference in reporting across settings, such as fear of discrimination, could cause patients with lower levels of comfort with English to report English as “principally spoken” despite preferences for non-English communication. Communication in English with a patient who might be served more comfortably with a non-English language may affect patient care from a practice standpoint and skew estimates from a data perspective. Further, disparities across racial and ethnic groups among proficient English speakers may allude to communication barriers beyond formal language, possibly to include understanding of medical terminology, proficiency in navigating the health care system, or health literacy [11]. 

Estimates of risk-adjusted mortality and obstetric trauma among API language subgroups are examples with clearer implications. Studies using disaggregated API ethnicities and languages found large variations in health care quality, access, utilization, and outcomes [24,29,30,31,32,33]. The findings of this study, which demonstrate that aggregation masks significant subgroup variation, support the collection and reporting of more granular data for research, evaluations, and targeted interventions. Although API-speaking inpatients collectively displayed a similar rate of stroke mortality compared with White patients, patients who spoke Japanese demonstrated a significantly higher rate of stroke mortality compared with White patients. More information on country of nativity or more granular data on race/ethnicity would be needed to decipher if this difference is attributable to Japanese nativity, language, or both. Even when API individuals are the population of interest in research studies, they are often aggregated because of limitations in sample size [23,34,35]. 

This study demonstrates that, even with a large number of records from a state with a relatively large proportion of self-reported API language speakers, the need remains for more detailed data to disentangle racial and linguistic influences among subgroups. The demographic characteristics of immigrants to the United States, particularly in some geographic regions, may also reflect bias. For example, API patients in this study were from communities with the highest incomes. Data on this topic from additional states and countries would reveal if results from the United States can be generalized to other countries that use English as the primary language or to countries with other primary languages.

Collection and reporting of language data are central to current and upcoming federal funding policies aimed at improving care to patients facing language barriers in the United States. The National Standards on Culturally and Linguistically Appropriate Services of 2001 [36] require competent linguistic assistance and consideration of a patient’s language preference for receipt of federal funds. In 2011, the Department of Health and Human Services published principal language data collection standards for federally conducted surveys or supported health care programs under the Affordable Care Act [37]. The document includes details on English proficiency and languages other than English spoken at home. Hospital collection of a patient’s language in a standard format will expand with the Medicare and Medicaid Electronic Health Record Incentive Programs that provide a financial incentive to hospitals for achieving “meaningful use” [38]. The Stage 1 meaningful use requirements include “preferred language” (the language by which the patient prefers to communicate) as one of the patient demographic data elements to be collected. Although California is one of a few states that collect information on the patient’s language in its statewide hospital discharge abstract data, recent changes to the hospital Uniform Bill standard [39] will facilitate additional states receiving this information from hospitals. 

### 4.3. Interpretation Services in the Health Care Setting 

Using professional interpreters in the hospital setting improves the quality of care for patients with limited English proficiency, and federal standards in the United States mandate the provision of such services. Interpreters may be provided through in-person or telecommunication models. However, previous studies using data from California hospitals suggest that interpreters are rarely used [40]. We could not determine whether interpreters were used during encounters in the present study, because this information is not captured by administrative data. Globally, as wide-scale migration becomes more common, interpretation and other services in health care and other settings that are geared toward individuals who speak a minority language will increase in importance. Future research examining the use of interpretation services alongside other patient and hospital characteristics would help to illuminate whether speaking a language other than English is a predictor of inpatient mortality for common conditions as well as obstetric trauma.

### 4.4. Limitations

This study uses administrative data and employs the AHRQ IQIs and PSIs, which are based on limited clinical information and represent a subset of possible hospital outcome measures. As discussed previously, administrative data do not provide information about clinical services such as interpreters, and the large sample size may have resulted in statistically significant results that are not necessarily clinically significant. However, all differences were greater than 10%, and even small differences in mortality and safety measures are clinically important. 

The reported rates reflect events taking place within the hospital setting, so deaths outside of the hospital are not captured. There was no adjustment for patient characteristics such as socioeconomic status, and we did not examine possible hospital selection effects to determine whether subpopulations are more likely to seek care or be sent to lower or higher quality hospitals. The data included in this study are limited to the state of California; therefore, findings may not be generalizable. This study is based on administrative data that rely on the self-report of the patient’s principal language spoken. Therefore, some amount of reporting bias may exist, particularly if a patient perceives “English” as a more socially acceptable response. In addition, the accuracy of the language data is contingent upon hospitals engaging patients to self-report their spoken language rather than assessment by staff, which could also bias the language categories. 

## 5. Conclusions 

This study represents the largest language study of inpatient mortality and obstetric trauma among inpatients to date. We analyzed data from over 3 million inpatients, including more than 70,000 Asians. The API sample was large enough to allow us to analyze seven subgroups, which increased the granularity of these data beyond what has been shown in most previous studies. In this population of California inpatients, speaking a non-English language and having a non-White racial/ethnic background did not place patients at a higher risk for inpatient mortality for the selected study conditions compared with English speakers; the exception was Japanese-speaking patients, who had higher stroke mortality. However, patients speaking an API language or having an API racial/ethnic background demonstrated higher obstetric trauma risk overall, whereas only Spanish-speaking Hispanic patients were at higher risk than their English-speaking counterparts. These differences should be examined further through multivariate analyses that address the confluence of other patient, primary payer, and hospital characteristics or procedures. Our results highlight the need for more research that examines the types of hospital outcomes affected by language and disentangles racial/ethnic and language effects while controlling for other explanatory factors. Findings imply that the strategic collection of more granular language data—particularly for Asians—as well as information on language preferences, proficiencies, access to interpreters, and health literacy may be warranted to better understand potential language variations.
